# Range expansion underlies historical introgressive hybridization in the Iberian hare

**DOI:** 10.1038/srep40788

**Published:** 2017-01-25

**Authors:** João P. Marques, Liliana Farelo, Joana Vilela, Dan Vanderpool, Paulo C. Alves, Jeffrey M. Good, Pierre Boursot, José Melo-Ferreira

**Affiliations:** 1 CIBIO, Centro de Investigação em Biodiversidade e Recursos Genéticos, InBIO Laboratório Associado, Universidade do Porto, 4485-661 Vairão, Portugal; 2 Departamento de Biologia, Faculdade de Ciências do Porto, 4169-007 Porto, Portugal; 3 Division of Biological Sciences, University of Montana, 32 Campus Drive, Missoula, MT 59812, USA; 4 Institut des Sciences de l’Évolution, Université Montpellier, CNRS IRD, Case Courrier 063, Place Eugene Bataillon, 34095 Montpellier, France

## Abstract

Introgressive hybridization is an important and widespread evolutionary process, but the relative roles of neutral demography and natural selection in promoting massive introgression are difficult to assess and an important matter of debate. Hares from the Iberian Peninsula provide an appropriate system to study this question. In its northern range, the Iberian hare, *Lepus granatensis*, shows a northwards gradient of increasing mitochondrial DNA (mtDNA) introgression from the arctic/boreal *L. timidus,* which it presumably replaced after the last glacial maximum. Here, we asked whether a south-north expansion wave of *L. granatensis* into *L. timidus* territory could underlie mtDNA introgression, and whether nuclear genes interacting with mitochondria (“mitonuc” genes) were affected. We extended previous RNA-sequencing and produced a comprehensive annotated transcriptome assembly for *L. granatensis*. We then genotyped 100 discovered nuclear SNPs in 317 specimens spanning the species range. The distribution of allele frequencies across populations suggests a northwards range expansion, particularly in the region of mtDNA introgression. We found no correlation between variants at 39 mitonuc genes and mtDNA introgression frequency. Whether the nuclear and mitochondrial genomes coevolved will need a thorough investigation of the hundreds of mitonuc genes, but range expansion and species replacement likely promoted massive mtDNA introgression.

Hybridization and genetic introgression between closely related species is a relevant evolutionary process that is widespread in nature^1^. Particularly frequent are cases of mitochondrial DNA introgression, often with apparently little or no nuclear DNA introgression, such as in elephants^2^, hares^3^ or chipmunks^4^ (see Toews *et al*.^5^ for a review). This raises important questions related to the demographic or adaptive processes underlying such a common phenomenon^5^. Many of the described cases of introgression may have resulted from range shifts and population replacements, which have presumably accompanied interactions between species during the drastic climate changes accompanying Pleistocene glacial cycles^6^. Understanding the causes and consequences of such introgression is of great interest to evolutionary biology^7^, but is an exceptionally difficult endeavor. On the one hand, invasion and range replacement with hybridization may promote massive gene flow from the resident species into the invading species in its newly colonized territories. This demographic model predicts that high frequencies of introgressed variants are caused by their fixation on the invasion front due to genetic drift, as suggested both by simulated^8^ and empirical data^9^. On the other hand, incorporating variants of a locally adapted resident species could facilitate colonization of new niches by the invading one^10^. The Pleistocene glacial oscillations strongly induced these interactions, by forcing species to change their ranges and promoting novel secondary contacts during the process^11^,^12^.

Hares from Western Europe illustrate this range shift phenomenon, and appear as ideal models to study the causes and consequences of historical gene flow during the range shifts of the Pleistocene. The fossil record shows that the distribution of species greatly changed during glacial oscillations. The mountain hare, *Lepus timidus*, is currently distributed in the northern Palearctic and in some isolated populations such as Ireland, Scotland, Poland and the Alps, but fossils from southern France or northern Iberian Peninsula show that it inhabited southern Europe during the Pleistocene^13^. The three extant species of the Iberian Peninsula, *L. granatensis, L. europaeus* and *L. castroviejoi*, now show high frequencies of mitochondrial DNA (mtDNA) haplotypes introgressed from *L. timidus*, acquired through hybridization at the end of the last glacial period before the latter went locally extinct^14^.

In the Iberian hare, *Lepus granatensis* (a species that is distributed across the Iberian Peninsula, except in the northernmost part) mtDNA introgression is strongly structured, being absent from the south and increasing in frequency towards the north. This pattern may be compatible with a northwards expansion of the species after the last glacial maximum, replacing and hybridizing with *L. timidus,* and spreading the traces of introgression to the north^15^,^16^. However, the demographic history of *L. granatensis*, which could account for patterns of introgression, has not yet been inferred. Ecological niche modeling partially supports the northwards range expansion scenario, showing that areas where mtDNA introgression is found are those with highest habitat favorability for *L. timidus* at the last glacial maximum^17^. Still, the highest climatic favorability for *L. granatensis* in the same period is more dispersed across the Iberian Peninsula^18^. Moreover, evidence from nuclear markers (10 autosomal, two X-linked and one Y-linked) suggest that introgression is rare (but see pattern of another X-linked locus^19^) and occurs all over the range of *L. granatensis*^3^. This contrast questions the plausibility of a purely demographic scenario as accounting for the structure of mtDNA introgression; a striking pattern that is also repeated in the two other hare species of the Iberian Peninsula, *L. europaeus* and *L. castroviejoi*^20^. Considering the strong functional role of mtDNA-encoded peptides in energy metabolism, their close interactions with nuclear encoded peptides, and evidence for positive selection during the evolution of the *L. timidus* mtDNA lineage^21^, cytonuclear coevolution and co-introgression in the northern range of *L. granatensis* remains a strong alternative hypothesis.

In this work we put together published and newly obtained RNA-sequencing data from *L. granatensis*, reconstruct a high quality transcriptome for the species, and then genotype a subset of 100 ascertained single nucleotide polymorphisms (SNPs). Using these data, we i) test for correlation between variation at nuclear genes with mitochondrion-associated functions and mitochondrial DNA introgression, and ii) infer the demographic history of the species.

## Results

### RNA sequencing, transcriptome assembly and functional annotation

**Figure 1:**
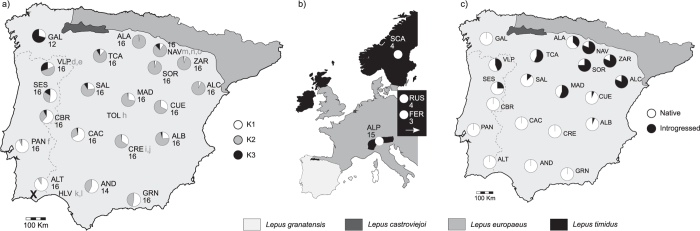
Geographic distribution of four hare species in the Iberian Peninsula (**a,c**) and Western Europe (**b**) (approximate distributions were based on Mitchel-Jones *et al*.^57^). (**a**) Localities sampled for *L. granatensis* (see Supplementary Table S1 for a detailed description); numbers indicate sample size, pie charts the proportion of STRUCTURE assignment to each of 3 clusters using the LOC prior and the complete SNP dataset (100 loci); the “X” marks the inferred origin of the range expansion; grey letters indicate specimens for which RNA was sequenced and used to build the transcriptome (“d-n” from Cahais *et al*.^22^ and Gayral *et al*.^23^, and “o” from this work). (**b**) Localities sampled for *L. timidus*, indicating the sample sizes; two sampling localities are not shown on this map (RUS – Russia; and FER – Far East Russia). (**c**) Proportion of the mitochondrial DNA lineages, native *L. granatensis* or introgressed from *L. timidus*, in the genotyped samples. Maps were generated in vectorial format using Inkscape v0.91 (https://inkscape.org).

The cDNA library prepared from liver and kidney tissues of *Lepus granatensis* specimen “o” (Fig. 1), harvested in Navarra during the hunting season, was sequenced in 1/12^th^ of an Illumina HiSeq 2000 lane, produced a raw output of 14,645,969 100 bp paired-end reads. Adapter removal and quality trimming resulted in 13,052,770 paired-end reads for a total of 2,496,629,898 bp. Previously published data from 10 other *L. granatensis* specimens^22^,^23^ (Fig. 1 and Supplementary Table S1; a total of 42,529,555 single-end 100 bp reads) were added to the dataset, which was used for *de novo* transcriptome assembly. A total of 54,838 contigs, ranging from 224 to 12,481 bp were obtained, which was reduced to 50,580 contigs after removing redundancy considering sequence similarity (Table 1). The transcriptome was further filtered by retaining only contigs with reciprocal best blast hit annotation against the rabbit genome (21,833 contigs) and/or containing a predicted open reading frame (ORF), resulting in a final assembly with 24,608 contigs, an N50 of 1,724 bp and a total length of 26,161,714 bp (Table 1). When compared with the available transcriptome produced by Cahais *et al*.^22^, the addition of relevant paired-end data caused important improvements in several statistics of the reconstructed assembly, such as unfiltered N50 (1334 vs. 909) and reference coverage (32% vs. 23%) (Supplementary Table S2).

To predict the potential functions of the assembled transcripts, the retained unigenes were aligned with various protein databases in addition to the *O. cuniculus* protein collection: NCBI, SwissProt, Gene Ontology, InterProScan and KEGG. The resulting functional annotation proportions are depicted in Supplementary Table S3, and the most represented GO terms shown in Supplementary Fig. S1.

### SNP inference and genotyping

After filtering for missing data and minimum allele frequency, a total of 3,532 SNPs were inferred in the eleven sequenced specimens. 3,402 of these SNPs occur in 1,196 genes annotated through reciprocal best blast hit with rabbit cDNA and peptide collection, while 130 were present in unannotated contigs. After alignment with rabbit genomic sequences, 1,119 genes remained, on which selection of SNPs for further genotyping was done in three distinct classes: A) randomly selected, avoiding only the selection of SNPs occurring in the same gene (44 SNPs); B) laying in nuclear genes involved in mitochondrial functions and ordered according to differentiation between the RNA-seq samples from regions with and without mtDNA haplotypes of *L. timidus* origin (see Fig. 1c) (63 SNPs with F_ST_ ranging between 0.6641 and 0); and C) with the same criteria as B but no functions on mitochondria (44 SNPs with F_ST_ ranging between 0.7869 and 0.3803).

Four Sequenom multiplexes were constructed with the 151 selected SNPs, which laid on 133 genes (redundant SNPs for genes of category B were included). These loci were genotyped in 317 *L. granatensis* (which included the 11 sequenced specimens) and 30 *L. timidus* (see Fig. 1 for sampling localities). Population genetic analyses were performed using 314 *L. granatensis* specimens that were organized in sample localities with more than 12 individuals each. After filtering out loci that were invariant, had more than 20% missing data, had inconsistent genotype composition for the 11 RNA-sequenced specimens (used as controls in the genotyping), or laid on the same gene/contig, 100 SNPs remained for the population genetics analyses (31, 39 and 30 from categories A, B and C respectively). Minimum allele frequency in the genotyped loci ranged between 13 and 50%. All SNPs were unlinked and in Hardy-Weinberg proportions.

### Correlation with mtDNA introgression

First we tested whether variation among *L. granatensis* populations was correlated with the prevalence of mitochondrial DNA introgression from *L. timidus*. Such correlation would suggest coevolution of the nuclear genome in response to the prevalence of the alien mitochondrial genome. This would be of particular interest if it concerned gene(s) of category B, involved in mitochondrial functions, as compared to those of category C, not involved but chosen with the same ascertainment method. We considered mtDNA introgression frequency as an environmental variable and tested its correlation with variations of allele frequencies among populations, using the Bayenv2 method^24^. We used the set of random SNPs to determine the covariance of allele frequencies due to population history, and then tested loci in categories B and C for association with mtDNA introgression prevalence. No significant associations were found.

### Population structure and range expansion

We then sought to infer the population differentiation history over the species range, for which a set of presumably neutrally evolving loci is needed. Loci in category A (randomly selected) can be considered appropriate in this respect (note also that these SNPs either represent synonymous substitutions or lay in a non-coding portion). Loci in the two other categories were chosen with an ascertainment method that could introduce a bias towards loci with a certain differentiation pattern. However, although this ascertainment method was meant to increase the chance to find genes whose evolution was correlated to mitochondrial DNA introgression, we found no sign of such parallelism in any of the analyzed loci (see above). Furthermore, we estimated the differentiation between mtDNA introgressed and non-introgressed populations on our much larger sample in an analysis of molecular variance (using an AMOVA^25^). We found no significant difference in the distribution of the Φ_CT_ statistic between category A and either category B or C SNPs (Mann-Whitney test on pairs and Kruskal-Wallis on all three; mean Φ_CT_ of 0.01623, 0.0149 and 0.0155 for classes A, B and C respectively). Therefore, the results based on the 11 RNA-sequenced samples were very poor predictors of the genetic structure of the species, and no ascertainment bias towards SNPs with the targeted differentiation pattern seemed to prevail in our SNP dataset.

Using BayeScan^26^, we inspected whether some of our loci showed any outlier pattern of differentiation among sampling localities. We found four loci with outlier patterns, three showing decreased differentiation (negative alpha; SNP033, SNP121 and SNP145) and one showing increased F_ST_ (positive alpha; SNP119). However, this method is expected to produce many false positives in situations of range expansion^27^, and this result should thus be interpreted carefully.

Below we present results based on the whole set of SNPs to infer population structure and history. However, all tests were also performed separately on the randomly selected subset (31 SNPs) and the set of non-outlier loci from the BayeScan analysis (96 SNPs), and produced similar results (see complete results in Supplementary Tables S4 and S5 and Supplementary Figs S2–4).

**Figure 2:**
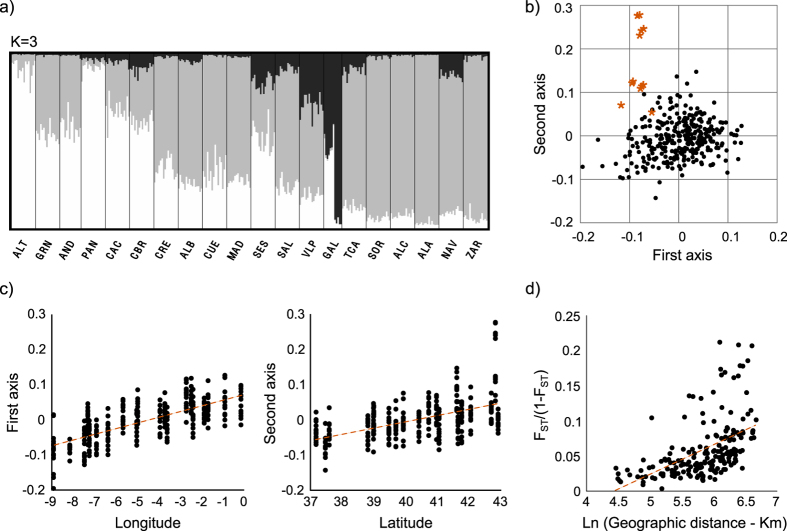
Organization of genetic diversity in *Lepus granatensis* from the analysis of 100 SNP loci (see Supplementary Figs S2–S5 for a complete description of the results obtained with several subsets of the dataset): (**a**) Structure plots with individual assignment to 3 clusters, as determined using Evanno’s delta K method, and using the sample locations as priors of the admixture model; population codes as in Fig. 1; (**b**) coordinates of samples on the first two axes of variation determined with a Principal Component Analysis (PCA) (stars correspond to specimens from Northwestern Iberian population GAL; see Fig. 1); (**c**) Correlation between the first two PCA axes of variation and geographical coordinates of sample localities (Spearman rank correlation, p = 0.00 for both analyses; dashed line indicates a linear regression trendline); (**d**) correlation between genetic differentiation and geographic distance among pairs of populations (Spearman rank correlation, p = 0.00; dashed line indicates a linear regression trendline).

We tested for population subdivisions using the Bayesian methods implemented in STRUCTURE^28^. Partitioning into three clusters was the most favored hypothesis (Supplementary Table S4), with clusters being predominant in different geographic regions: K1 in the Southwestern part, K2 in the central and Northeastern part and K3 in the Northwestern part, around population GAL (Fig. 1a). Most localities appeared admixed between at least two clusters and, apart from a possible distinct genetic cluster in the Northwest (population GAL), a geographical gradient of individual assignment was apparent (Fig. 2a). This coincided with a pattern of isolation by distance (Fig. 2d, Spearman ranked correlation one-tailed P-value = 0.00 in the Mantel test of correlation between genetic and geographic distance). A principal components analysis confirmed these results and showed strong correlation of the two first axes of variation with longitude and latitude (Spearman ranked correlation one-tailed p-value = 0.00; Fig. 2b and c). Again, specimens from GAL were suggested to have some level of genetic differentiation from the rest (stars in Fig. 2b).

These patterns of weak clustering and correlation of genetic and geographic distances could reflect isolation by distance in an equilibrium population, vast admixture between historically differentiated populations, or past expansion over the species range. To explore the two latter possibilities, we used the method of Peter and Slatkin^29^ to infer range expansion and its direction. The secondary contact hypothesis predicts independent and geographically convergent expansions in different areas (corresponding to the areas of prevalence of the different STRUCTURE clusters), while the range expansion hypothesis predicts a single expansion over the species range. We thus applied the test of expansion species-wide, as well as separately on subsets of populations (regions) grouped according to the cluster they were assigned to in majority (region R1 in the Southwest where K1 was predominant, R2 in the Centre and Northeast where K2 was the most frequent, and R3 in the Northwest where K3 was the major cluster, see Fig. 1a). Taking all three regions together, a signal of range expansion was inferred, with an origin near locality ALT (Fig. 1a, P < 0.05) (see detailed results for all analyzed datasets in Supplementary Table S5). Allowing for the possibility of multiple origins, significant support for range expansion was only obtained when considering R1 and R2 together with origin again near ALT (P < 0.001), and when taking R2 alone, with origin near CRE (P < 0.001), thus also suggesting expansion from South to North. In summary, these results are neither compatible with a model of isolation by distance, nor with a model of secondary contact between the K1 and K2 clusters. They are compatible with a global range expansion from Southwest Iberia into the rest of the Peninsula. The south-north signal of expansion is most pronounced in the northern half of the Peninsula, the region where mitochondrial introgression from *L. timidus* prevails. The direction of expansion parallels the gradient of mtDNA introgression, with higher introgression in the direction of expansion. The Northwestern populations (around GAL) may not result from this global expansion, and are not affected by mitochondrial introgression^14^. There appears to have been an isolated pocket in this region, which secondarily admixed with the other populations. Similar conclusions are drawn from the analysis of the randomly selected loci and the dataset removing potential F_ST_ outliers (Supplementary Table S5).

## Discussion

Extensive mitochondrial DNA introgression between species is very common in animals and cytonuclear co-evolution is often proposed as a likely explanation for these striking patterns^5^. In addition to providing further insights into the evolutionary history of hares, our study provides a relatively rare test of the alternative hypotheses that may account for mtDNA introgression.

We first produced a *de novo* assembly and annotation of the transcriptome of *L. granatensis,* and improved its quality when compared to a previous study^22^. The availability of RNA-sequencing from 11 individuals distributed across the range of the species allowed discovering numerous SNPs, which is a valuable resource for future work on this and related species. We could successfully validate a subset of the discovered SNPs on a large sample of individuals and then used them to address the question of general interest related with the causes and consequences of massive mitochondrial DNA introgression. One of the first objectives of our study was to discover nuclear genes involved in mitochondrial function, i.e. interacting with the mitochondrial genome or its products, which evolved under the influence of the massive prevalence of an alien mitochondrial genome in some populations. This is a difficult statistical question because shared population history can cause correlations between allele frequencies at different loci that are not related with adaptation or coevolution. We therefore used a method that accounts for such correlations and removes their confounding effects, and treated mitochondrial DNA introgression frequency as an environmental variable, but found no correlation. We attempted to increase the chance of finding such genes by selecting SNPs with apparent high differentiation between mitochondrial DNA introgressed and non-introgressed regions based on the initial set of 11 sequenced specimens. We also applied the same ascertainment bias to genes not involved in mitochondrial metabolism as a control. However, enrichment on this basis was unsuccessful because the allele frequency estimates derived from the small ascertainment sample were shown to be very poor predictors of the species-wide patterns. This weakened our ability to find evidence for coevolution of the nuclear genome accompanying the massive mtDNA introgression. We also did not find any case where the SNP variant present in *L. timidus* is rare in the south and predominant in the northern range of *L. granatensis* where the mtDNA haplotypes of the former are frequent, which suggests no cytonuclear co-introgression among our genotyped loci. A more thorough investigation of cytonuclear coevolution in this system is therefore needed, including all genes potentially interacting with the mitochondrial genome and its products. Interest in this question is revived by recently reported suspicion of interspecific cytonuclear incompatibilities in a mammal species^30^.

Given the lack of association between mtDNA and our nuclear SNPs, we were able to consider the chosen loci as neutral to make inferences on population history and demography. We obtained similar results when using both the randomly selected loci and the dataset removing potential F_ST_ outlier loci (that could actually result from the range expansion we also inferred^27^), which suggests that the complete SNP set is appropriate for this purpose. The relative demography of interacting species is an important determinant of the rate and direction of introgression between them^8^. Importantly, it can explain unequal levels of introgression along the genome and geographic gradients of introgression, similar to patterns resulting from introgression promoted by natural selection along an environmental gradient. Hares from the Iberian Peninsula have been widely affected by introgression from the arctic/boreal *L. timidus*, but the reconstruction of demographic histories can only be performed in the receiver species, because the donor is locally extinct. In this work, we provide the first insights into the population history of *L. granatensis*, which can be used to interpret patterns of historical introgression of *L. timidus* origin.

Interpreting geographical variations of allele frequencies of particular loci as resulting from either selection or demographic and historical contingencies is notoriously difficult. This question can be addressed in different ways, depending on priors about species history and ecology and the function of the genes studied. Here we had a strong hypothesis of a south-north range expansion that could account for the geographically restricted and massive mitochondrial DNA introgression^3^,^15^,^16^,^19^. The signal of range expansion is clear in the Northern part where introgression is present, and weak considering only the South of the Peninsula, at the origin of range expansion and where mitochondrial introgression is absent (Fig. 1; Supplementary Table S5). It follows the direction expected to have created a South-North gradient of increasing introgression, due to allele surfing and potentially repeated introgression along the invasion and hybridization front^8^, as evidenced in *L. europaeus*, in its Iberian range^16^. However, the sample of loci we used to infer range expansion is relatively small^29^ and should be treated as preliminary. Interestingly, we also found that the Northwestern populations appear to have evolved separately and not to have been involved in the South-North expansion that underlies the hybridization events. Concordantly, they are not affected by mitochondrial DNA introgression (Fig. 1).

Our results provide support for the important role of purely demographic processes in promoting massive mitochondrial DNA introgression, and suggest that this phenomenon may explain the common nature of mtDNA reticulation. Even though the phenomenon is expected to particularly affect regions of the genome linked to the least dispersing sex^8^ (which in mammals are often females), similar gradients of introgression may occur along the genome. Therefore, understanding whether the patterns of mtDNA introgression in the affected species is generally accompanied by concomitant frequencies and distribution of introgressed nuclear DNA variants, as a result of the very same demographic dynamics, depends on a thorough inspection of the genome, which in most of the model systems for mtDNA reticulation has been poorly explored (Good *et al*.^4^ showed that 95% of the models of cytonuclear discordance reviewed by Toews and Brelsford^5^ analyzed 20 or less nuclear markers). In addition, evolutionary histories are complex and we cannot dismiss the possibility that massive introgression had selective consequences on the nuclear genome, either by promoting coevolution of some mitochondrion nuclear genes or favoring co-introgression of such genes (hypotheses we had little power to test due to the small number of genes screened here). Introgression of locally adapted genes may facilitate range replacements and co-contribute to patterns of widespread reticulation. Again, only genome-wide surveys will allow addressing this question appropriately.

## Methods

### RNA-sequencing, de novo transcriptome assembly, validation and annotation

Liver and kidney samples from one *Lepus granatensis* (specimen “o” in Fig. 1) harvested in Navarra, Spain, during the hunting season were collected shortly after the death of the animal and immediately placed in RNAlater and then stored at −20 °C until RNA extraction. Grinding of the tissues was done separately, with liquid nitrogen and a ceramic mortar and pestle. RNA extraction was then performed using the QIAGEN RNeasy^®^ Mini Kit following the manufacturer’s protocol. The concentration of the RNA extracts and RNA Integrity Number (RIN) were estimated using a Bioanalyzer 2100 (Agilent Technologies). The RNA extracts of the two tissues were then pooled in equal proportions, cDNA libraries were produced using the TruSeq RNA Sample Preparation Kit (Illumina) and the fragment size distribution checked using a Bioanalyzer 2100 (Agilent Technologies). The library was then pooled in equimolar proportions with cDNA libraries produced for other purposes and sequenced in 1/12^th^ of one lane of an HiSeq2000 at the QB3 Computational Genomics Resource Laboratory (CGRL), University of California, Berkeley, producing 100 bp paired-end reads. Low quality reads were removed using CASAVA-1.8 FASTQ Filter, adapter sequences were identified and removed using Cutadapt v1.3^31^ and reads were trimmed for quality using Trimmomatic v0.32^32^ with options LEADING:5 TRAILING:5 SLIDINGWINDOW:4:15 MINLEN:20. Previously published transcriptome single-end sequence reads from 10 *L. granatensis* specimens^22^,^23^ (data available from ftp://ngsisem.mbb.univ-montp2.fr/phylogenie_et_evolution_moleculaire/pub/popphyl/reads/Lepus_granatensis/) were added to the dataset (see geographic distribution of the 11 sampled specimens in Fig. 1a).

*De novo* transcriptome assembly was constructed using Trinity v.2.1.0^33^ with default parameters and data from the 11 specimens (single-end data from 10 specimens and paired-end data from 1 specimen). Large amounts of chimeras and poorly supported contigs can arise during the assembly process. Therefore, in order to evaluate the quality of the produced assembly, sequenced reads were mapped back to the reference assembly and quantitative and qualitative measures were applied using TransRate^34^. A redundancy filter was then applied using CD-HIT-EST v 4.6.4^35^ to remove contigs with more than 95% similarity. To evaluate the transcriptome completeness, cDNA and peptide collections of *Oryctolagus cuniculus* (European rabbit) were downloaded from ENSEMBL (http://www.ensembl.org/info/data/ftp; release 2.0.81), and used as reference (the average genomic divergence between rabbits and hares is expected to be around 5%^36^). TransDecoder v2.1.0^37^ was then used to predict the open reading frames and remove transcriptome noise (e.g. non-coding RNA, DNA contamination or erroneously assembled contigs). Filtered assembly was aligned against the rabbit transcriptome using Conditional Reciprocal Best BLAST^38^, which selects the reciprocal best hits from a bi-directional BLAST + alignment. The final transcriptomes consisted of unigenes with rabbit annotation and/or predicted open reading frame. Similar statistics were then obtained for the existing *L. granatensis* transcriptome produced by Cahais *et al*.^22^ to compare the quality and completeness of the assemblies.

Functional annotation was further performed to identify putative mRNA functions. Unigenes were additionally annotated using the blastx algorithm against Swiss-Prot^39^ and NCBI non-redundant protein databases^40^ applying an e-value cut-off of 1e-5. Additionally, NCBI non-redundant annotation output was incorporated on Blast2GO^41^ in order to perform a functional classification of the transcripts, through the assigning of Gene Ontology (GO) terms and prediction of metabolic pathways using the Kyoto Encyclopedia of Genes and Genome (KEGG)^42^,^43^. Complementarily, InterProScan^44^ was used to identify protein domains and the output was quantified.

### Inference of single nucleotide polymorphisms and genotyping

Mapping of the paired-end and single-end reads of the 11 *L. granatensis* specimens onto the *de novo* assembled transcriptome was performed using the bwa-mem v0.7.12 algorithm with default parameters^45^, and the resulting alignments were sorted using SAMtools v0.1.18^46^. SNP calling was performed using Reads2snp v2.0.64^23^,^47^ using a threshold of 20 for site and mapping qualities, a minimum coverage of 8X and a genotype probability > 0.95.

In order to guarantee an overall representation of the species range, only sites represented in at least 8 out of the 11 specimens and with a minimum of 4 specimens from each of the southern or northern halves of Iberia were retained for further analyses. In addition, low frequency variants were filtered out, retaining only sites with a minimum allele frequency of 0.2.

Spliced alignments of the assembled contigs with the rabbit genome were produced using sim4db v1.896^48^ in order to identify intron-exon boundaries. Only sequences with more than 90% identity and SNPs laying within the inferred alignment coordinates were retained for further analyses.

One first class of SNPs, selected randomly among independent contigs was identified for further genotyping – class A. Two additional classes were built, favoring SNPs with higher F_ST_ between regions with and without mtDNA introgression considering the 11 sequenced specimens. Among these SNPs, those laying on genes with functions related with the mitochondrion were ascribed to class B. These are candidates for potential coevolution or cointrogression with mtDNA. A third class was then considered among SNPs on genes with no mitochondria functions – class C – which served as control for SNPs from class B. Genes with functions in the mitochondria (mitonuc genes) were defined according to the InterMitoBase database list, based on human information^49^ (708 genes). Given that human gene annotation is extensively more complete than the rabbit one, gene codes were obtained using the human-rabbit 1:1 orthologs obtained from Biomart, or using blastx alignment against the human collection of peptides, when orthology information was missing.

Genotyping was performed using Sequenom MassARRAY at the Centre de Génomique Fonctionelle de Bordeaux, Plateforme Génome Transcriptome, Université Bordeaux 2. Preference was given for SNPs with at least one 100 bp flank with no polymorphism among the 11 sequenced specimens. In some cases, polymorphism in the flanking regions was taken into account with degenerate nucleotide symbols. Whenever possible two SNPs were genotyped at certain focal genes from SNP class B. After multiplex construction, the final selection included 151 SNPs laying in 133 genes (class A: 49 genes; class B: 42 genes; class C: 42 genes), which were genotyped in 317 *L. granatensis* (including the 11 sequenced specimens) and 30 *L. timidus* (see geographic distribution of sampling in Fig. 1).

### Genotype data analyses

Population genetics analyses were performed using 314 genotyped *L. granatensis* specimens that were organized in 20 sampling localities with at least 12 individuals each. Each genotyped locus was checked for conformation to Hardy-Weinberg proportions using genepop v4.2^50^. The same software was used to test for linkage disequilibrium between pairs of loci.

Given that the selections of SNPs from classes B and C were ascertained taking into account the geographic distribution of mtDNA introgression from *L. timidus*, we tested whether we could find any correlation using Bayenv2^24^ and treating introgression frequencies as an environmental variable. The method initially estimates a null covariance matrix from a set of randomly selected loci, and then assigns Bayes Factors to each SNP that measures whether allele frequencies co-vary with an environmental variable, over the null model of population structure. The set of randomly selected loci (class A) was used to estimate the covariance matrix based on 10 million iterations. Three replicate runs were performed to ensure the consistency of the estimates, and Bayes factors were averaged over the independent runs. The variable was normalized following the recommendation. A Bayes Factor > 3 was considered as indicative of correlation between the allele frequencies at a given locus and the variable.

We then checked whether the ascertainment based on differentiation between regions with and without mtDNA introgression based on the 11 sequenced specimens, used to select SNP classes B and C, indeed produced an enrichment of differentiation. This was tested using a locus-by-locus AMOVA^25^, as implemented in Arlequin v3.5^51^.

Also, we tested whether F_ST_ outliers could be found in our dataset, using the Bayesian method implemented in BayeScan v2.116. This method estimates whether subpopulation-specific allele frequencies differ from the common gene pool, as measured by an F_ST_ coefficient. The F_ST_ coefficient is decomposed in a locus-specific parameter, alpha, and a population-specific parameter, beta. A false discovery rate of 0.05 was used. Note however that this method is expected to produce many false positives when the true underlying demographic model is range expansion^27^.

Analyses of population structure were further performed using the randomly selected loci, the dataset without F_ST_ outliers and the full dataset. To investigate the partition of genetic diversity in *Lepus granatensis* the admixture model implemented in STRUCTURE 2.3.4^28^ was applied. A variable number of K populations, from 2 to 10, was considered and three replicate runs per partition with 1 million steps after a burn-in period of 500 000 were performed. The model considering sampling locations as prior information (LOC prior) was applied because it is expected to better detect shallow structure. Replicate runs were analyzed using CLUMPP v1.1.2^52^ and DISTRUCT v1.1^53^ was used to plot the results. The best number of populations, K, was inferred using Evanno’s delta K method^54^, as implemented in STRUCTURE HARVESTER^55^.

Possible partitions of genetic diversity in the dataset were further investigated using principal components analyses, as implemented in Eigenstrat^56^. In addition, the existence of a correlation between population differentiation and geographic distance was verified using the ISOLDE method implemented in Genepop^50^.

Finally, evidence for a range expansion and its putative origin was tested using the R library rangeExpansion^29^. This method estimates a directionality index that detects the clines of allele frequencies produced during range expansions. Range expansion was tested for the complete datasets and for subsets defined according to the STRUCTURE results, to detect possible multiple range expansions. Results were summarized and visualized using the summary and plot functions.

## Additional Information

**Accession codes:** Raw RNA-sequencing data newly obtained in this work is available at GenBank SRA with accession SRR4454540. The assembled transcriptome, table of inferred SNPs from RNA-sequencing data and the genotyping results are available at Dryad - doi:10.5061/dryad.g0hd8.

**How to cite this article:** Marques, J. P. *et al*. Range expansion underlies historical introgressive hybridization in the Iberian hare. *Sci. Rep.* **7**, 40788; doi: 10.1038/srep40788 (2017).

**Publisher's note:** Springer Nature remains neutral with regard to jurisdictional claims in published maps and institutional affiliations.

## Supplementary Material

Supplementary Information

## Figures and Tables

**Table 1:** Summary statistics of *L. granatensis de novo* transcriptome assembly, considering three filtering levels: raw assembly, removing redundancy and retaining contigs with ORF and/or reciprocal best blast hit onto rabbit transcripts and peptides.

	Raw	Non-redundant	ORF and/or blast hit
Number of contigs	54,838	50,580	24,608
Average contig length (bp)	800	761	1,063
Total length (bp)	43,877,813	38,480,389	26,161,714
Maximum contig length (bp)	12,481	12,481	12,481
Minimum contig length (bp)	224	224	224
N50 (bp)	1,334	1,247	1,724
Number of contigs > 1 kb	13,340	11,299	9,361
Proportion of contigs > 1 kb (%)	24.3	22.3	38.0
Reference Proteins with blast hit (%)	51	51	51
Reference coverage (%)	32	32	32
