# Supplementary material for: Range expansion underlies historical introgressive hybridization in the Iberian hare
Source: Sci Rep. 2017 Jan 25;7:40788. doi: 10.1038/srep40788 (PMC5264399; doi:10.1038/srep40788)
Supplement: Supplementary Information [file srep40788-s1.pdf]

SUPPLEMENTARY INFORMATION

Range expansion underlies historical introgressive hybridization in the Iberian hare

João P. Marques[1,2], Liliana Farelo[1], Joana Vilela[1], Dan Vanderpool[3], Paulo C. Alves[1,2], Jeffrey M. Good[3], Pierre Boursot[4], José Melo-Ferreira[1,2]

[1]CIBIO, Centro de Investigação em Biodiversidade e Recursos Genéticos, InBIO Laboratório Associado, Universidade do Porto, 4485-661 Vairão, Portugal.
[2]Departamento de Biologia, Faculdade de Ciências do Porto, 4169-007 Porto, Portugal.
[3]Division of Biological Sciences, University of Montana, 32 Campus Drive, Missoula, MT 59812, USA.
[4]Institut des Sciences de l'Évolution, Université Montpellier, CNRS IRD, 34095 Montpellier, France.

Table S1: Sampling localities, sample sizes for transcriptome assembly and SNP genotyping and proportion of mtDNA introgression in the genotyped samples.

Code	Locality	Latitude	Longitude	n Transcriptome	n Genotyping	Proportion Introgression
Lepus granatensis						
ALT	Alcoutim, Portugal	37.469978	-7.473078	-	16	0
ALA	Álava, Spain	42.910000	-2.698387	-	16	0.375
ALB	Albacete, Spain	38.994350	-1.858542	-	16	0.0625
ALC	Alcañiz, Spain	41.051037	-0.133537	-	16	0.8125
AND	Andaluzia, Spain	37.590711	-5.019765	-	14	0
CAC	Cáceres, Spain	39.471329	-6.370961	-	16	0
CBR	Castelo Branco, Portugal	39.924751	-7.241590	-	16	0
CRE	Ciudad Real, Spain	38.984829	-3.927378	2	16	0
CUE	Cuenca, Spain	39.690079	-2.381535	-	16	0.0625
GAL	Galicia, Spain	42.826070	-8.157443	-	12	0
GRN	Granada, Spain	37.177338	-3.598557	-	16	0
HLV	Huelva, Spain	-	-	2	2	-
MAD	Madrid, Spain	40.416775	-3.703790	-	16	0.5625
NAV	Navarra, Spain	42.695393	-1.676069	3	16	0.8125
PAN	Pancas, Portugal	38.809101	-8.918929	1	16	0
SAL	Salamanca, Spain	40.970104	-5.663540	-	16	0.125
SES	Serra da Estrela, Portugal	40.725407	-6.905594	-	16	0.25
SOR	Soria, Spain	41.764431	-2.463772	-	16	0.75
TCA	Tierra de Campos, Spain	42.049622	-4.976654	-	16	0.5625
TOL	Toledo, Spain	-	-	1	1	-
VLP	Valpaços, Portugal	41.608715	-7.310906	2	16	0.4375
ZAR	Zaragoza, Spain	41.648792	-0.889581	-	16	0.8125
Lepus timidus						
ALP	Alps	-	-	-	15	-
FAR	Far East Russia	-	-	-	3	-
RUS	Western Russia	-	-	-	4	-
SCA	Scandinavia	-	-	-	4	-

Table S2: Comparison of summary statistics of *L. granatensis de novo* transcriptome assemblies produced by Cahais *et al.* (2012)* and by this work.

Statistics	Cahais *et al.* 2011	This work	Variation (%)
Number of contigs	45151	54838	+18
Average contig length (bp)	657	800	+18
Total length (bp)	29676032	43877813	+32
Maximum contig length (bp)	13780	12481	-10
Minimum contig length (bp)	201	224	+10
N50 (bp)	909	1334	+32
Number of contigs > 1 kb	7526	13340	+44
Proportion of contigs > 1 kb (%)	16.7	24.3	+31
Reference Proteins with blast hit (%)	46	51	+10
Reference coverage (%)	24	32	+25

*Cahais, V. *et al.* Reference-free transcriptome assembly in non-model organisms from next generation sequencing data. *Molecular Ecology Resources* 12, 834-845 (2012).

Table S3: Number of unigenes resulting from functional annotation of the *L. granatensis* transcriptome with different protein databases.

	Number of unigenes	Percentage	200 ≤ Length < 1000	Length ≥ 1000
O. cuniculus genome (Ensembl, 2.0.81)	21833	88.7	13097	8736
SwissProt	21933	89.1	13028	8905
NCBI NR	22362	90.9	13383	8979
InterProScan	3580	14.5	1456	2124
KEGG	17072	69.4	8960	8112
Gene Ontology	16867	68.5	9599	7268
Annotated	22740	92.4	13679	9061
Non annotated	1868	7.6	1563	305

Table S4: Results of the Evanno method, indicating the mean likelihoods of the STRUCTURE runs for each tested K value.

K	Replicates	Mean LnP(K)	StD LnP(K)	Ln'(K)[1]	|Ln''(K)|[2]	DeltaK[3]
All						
1	3	-36867.4000	0.1000	-	-	-
2	3	-36530.2667	6.4003	337.1333	16.7667	2.619685
3	3	-36176.3667	3.6950	353.9000	216.000	58.456720
4	3	-36038.4667	10.6651	137.9000	71.4000	6.694763
5	3	-35829.1667	129.1684	209.3000	0.3000	0.002323
6	3	-35619.5667	13.7143	209.6000	149.6667	10.913150
7	3	-35559.6333	41.2723	59.9333	38.4333	0.931214
8	3	-35461.2667	26.0108	98.3667	332.1667	12.770320
9	3	-35695.0667	273.7944	-233.8000	533.7333	1.949395
10	3	-35395.1333	32.1808	299.9333	-	-
No F_{ST} outliers						
1	3	-35384.7333	0.0577	-	-	-
2	3	-35070.5333	9.0224	314.2000	17.000	1.884204
3	3	-34739.3333	6.1712	331.2000	187.5333	30.388620
4	3	-34595.6667	9.4691	143.6667	80.3000	8.480239
5	3	-34371.7000	77.1213	223.9667	29.6333	0.384243
6	3	-34177.3667	19.4526	194.3333	139.9000	7.191844
7	3	-34122.9333	4.5369	54.4333	88.2000	19.440650
8	3	-34156.7000	150.4748	-33.7667	104.6333	0.695354
9	3	-34085.8333	60.7023	70.8667	56.0333	0.923085
10	3	-34071.0000	116.5166	14.8333	-	-
Random						
1	3	-11491.3000	0.1000	-	-	-
2	3	-11340.2667	2.8042	151.0333	50.0000	17.830630
3	3	-11239.2333	2.8361	101.0333	85.5000	30.147280
4	3	-11223.7000	80.6865	15.5333	5.5333	0.068578
5	3	-11213.7000	70.4360	10.0000	83.0000	1.178375
6	3	-11120.7000	12.0926	93.0000	112.0667	9.267407
7	3	-11139.7667	18.5971	-19.0667	72.4333	3.894866
8	3	-11231.2667	94.6392	-91.5000	68.2000	0.720632
9	3	-11254.5667	50.2674	-23.3000	18.0667	0.359411
10	3	-11259.8000	115.1112	-5.2333	-	-

All – 100 loci; No F_{ST} outliers – removing the 4 FST outliers (retaining 96 loci); Random – using only the randomly selected loci (31 loci).

[1]Rate of change of the likelihood distribution (mean).

[2]Absolute value of the 2nd order rate of change of the likelihood distribution (mean).

[3]DeltaK = mean(|Ln''(K)|)/StD(Ln(K))

Table S5: Inferred origin of range expansion considering the complete or random datasets and population partitions according to STRUCTURE results.

			Origin of Range Expansion[4]			
Dataset[1]	K[2]	Partition[3]	Longitude	Latitude	Closest Sampling Locality	Significance[5]
All	3	R1+R2+R3	-7.942774	37.17734	ALT	*
All	3	R1+R2	-7.942774	37.17734	ALT	**
All	3	R1+R3	-8.918929	42.82607	GAL	-
All	3	R2+R3	-8.157443	42.91	GAL	-
All	3	R1	-8.918929	38.48452	PAN	-
All	3	R2	-4.120964	38.98483	CRE	**
All	3	R3	-	-	-	-
No F_{ST} outliers	3	R1+R2+R3	-7.942774	37.17734	ALT	-
No F_{ST} outliers	3	R1+R2	-7.942774	37.17734	ALT	*
No F_{ST} outliers	3	R1+R3	-8.918929	42.82607	GAL	-
No F_{ST} outliers	3	R2+R3	-8.157443	42.91	GAL	-
No F_{ST} outliers	3	R1	-8.918929	38.48452	PAN	-
No F_{ST} outliers	3	R2	-4.120964	38.98483	CRE	**
No F_{ST} outliers	3	R3	-	-	-	-
Random	3	R1+R2+R3	-8.918929	38.68593	PAN	-
Random	3	R1+R2	-8.918929	38.68593	PAN	**
Random	3	R1+R3	-8.918929	38.66385	PAN	-
Random	3	R2+R3	-8.157443	42.91	GAL	-
Random	3	R1	-8.918929	38.54131	PAN	-
Random	3	R2	-3.820206	39.81118	CRE	-
Random	3	R3	-8.157443	42.82607	GAL	-

[1]All 100 loci, removing the 4 FST outliers (retaining 96 loci) and using only the randomly selected loci (31 loci).

[2]Number of K clusters inferred with STRUCTURE; best K, as inferred using the Evanno deltaK method.

[3]Clusters of populations defined according to STRUCTURE assignment, by grouping sampling localities with predominant assignment to each K cluster.

[4]Origin of range expansion estimated with the rangeExpansion method.

[5]Significance of range expansion inference; *P<0.05, **P<0.001.

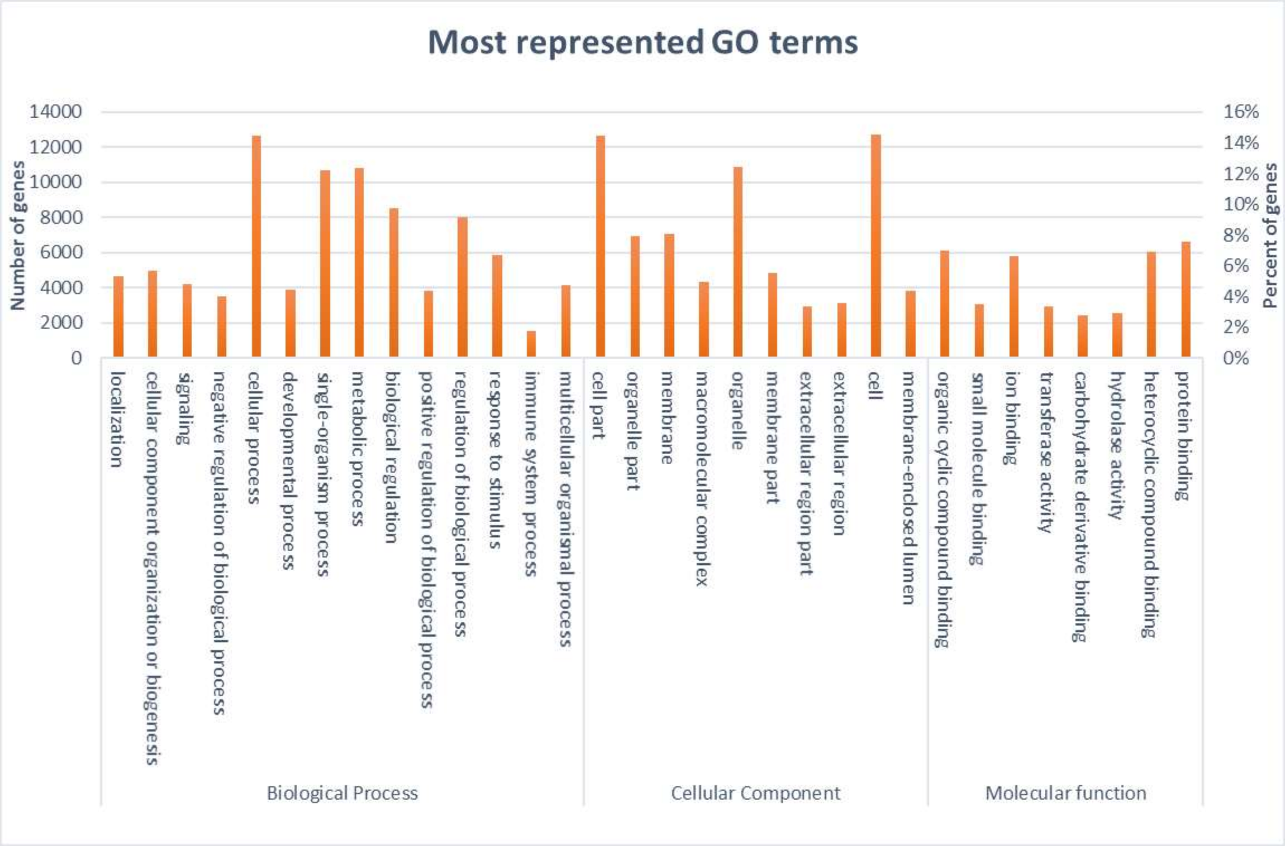

Figure S1: Most represented Gene Ontology (GO) terms in the *L. granatensis* transcriptome.

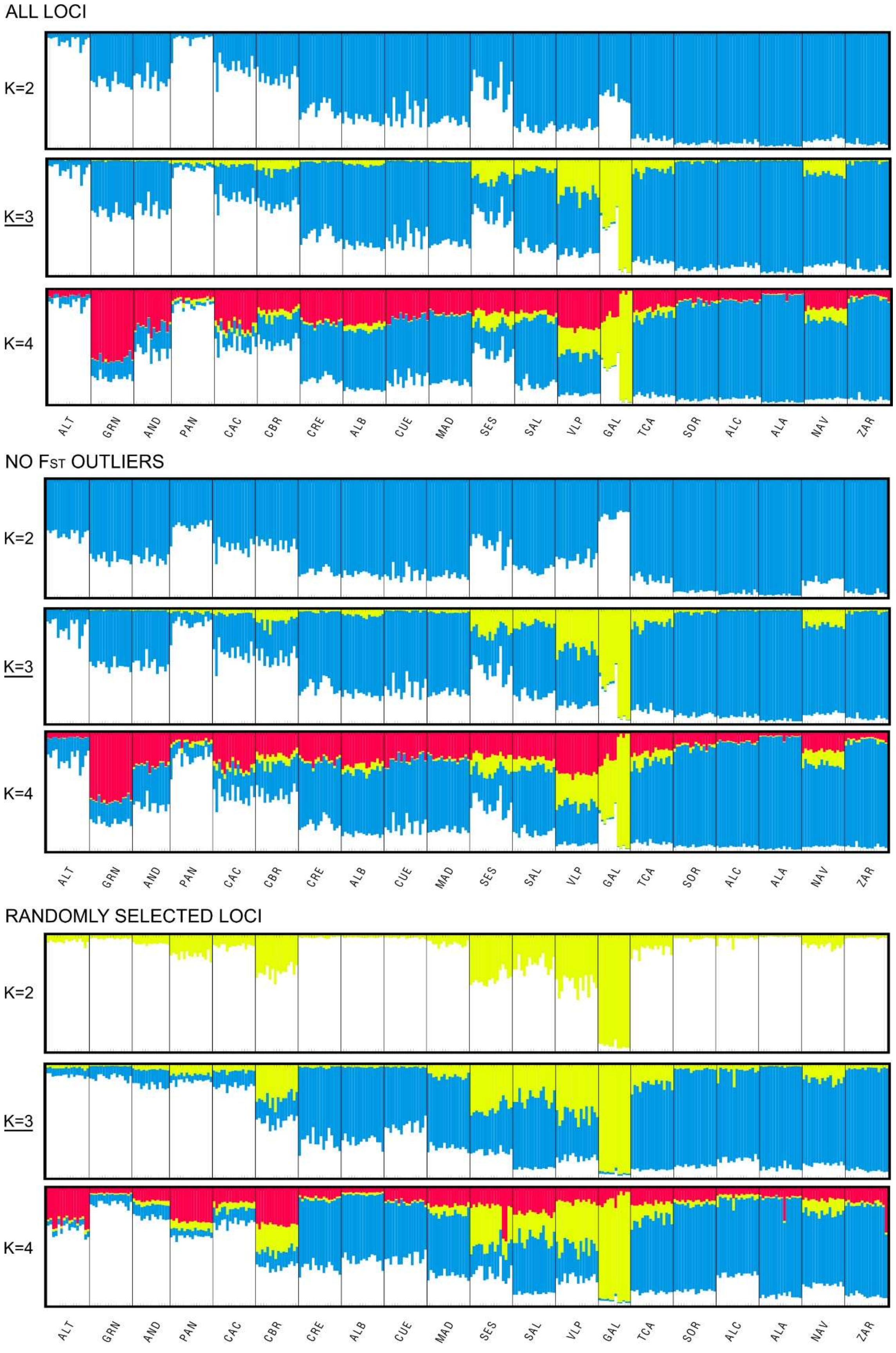

Figure S2: STRUCUTURE plots for K=2, 3 and 4 for analyses including all genotyped loci (100 SNPs), removing putative F_{ST} outliers (96 SNPs) and the randomly selected subset (31 SNPs). The best K, assessed using Evanno's delta K method, is underlined.

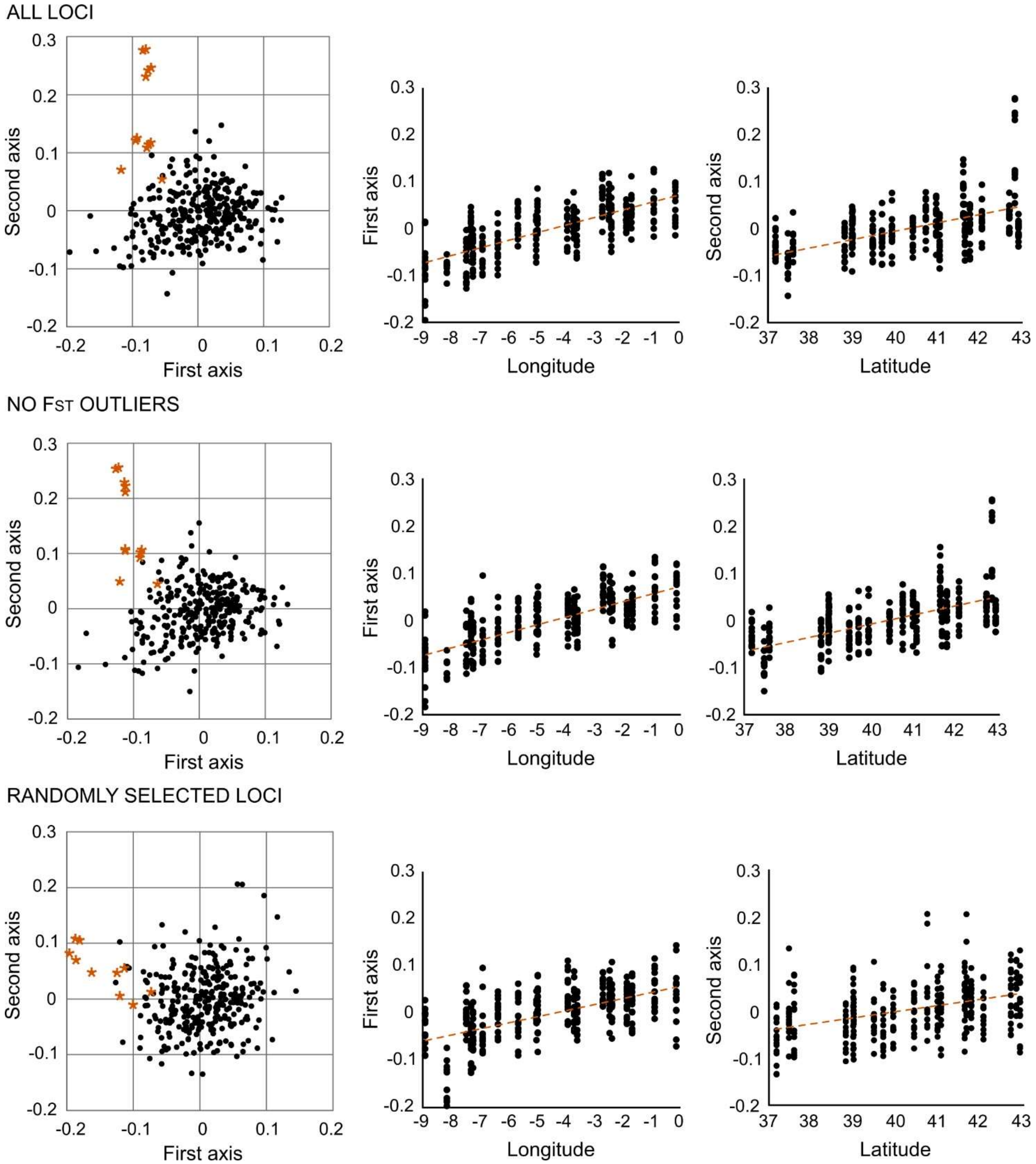

Figure S3: Principal component analysis (PCA) plots and correlation of the first two axes with longitude and latitude for the complete genotyped loci (100 SNPs), removing putative F_{ST} outliers (retaining 96 SNPs) and for the randomly selected subset (31 SNPs). Stars indicate samples from population GAL. All correlations are significant (Spearman rank correlation, p=0.00; dashed line indicates a linear regression trendline).

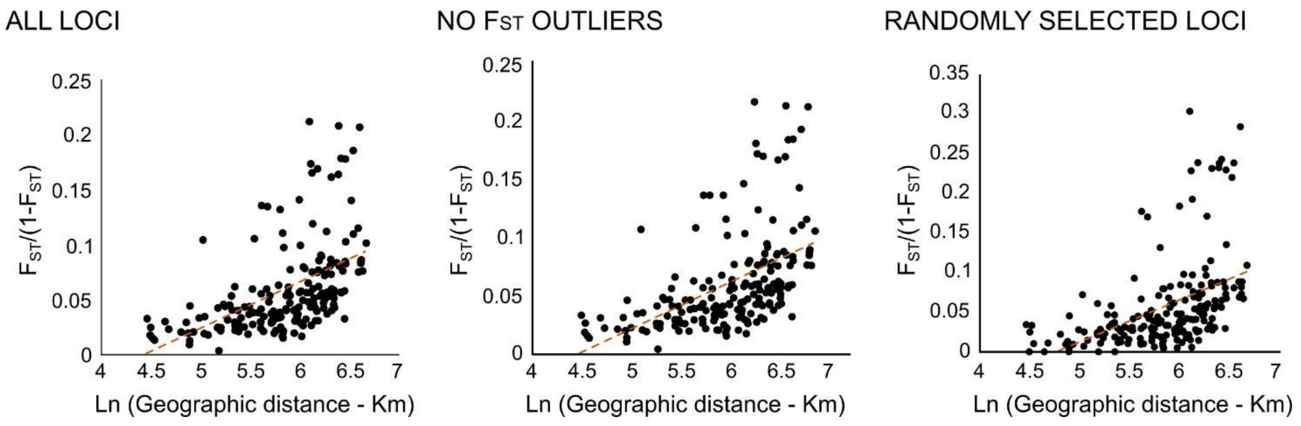

Figure S4: Correlation between genetic differentiation and geographic distance among pairs of populations for analyses conducted using all genotyped loci (100 SNPs), removing putative F_{ST} outliers (retaining 96 SNPs) and using the randomly selected dataset (31) (Spearman rank correlation, p=0.00 in both cases; dashed line indicates linear regression trendlines).
